# A Scoping Review of Interventions Targeting the Mental Health of Australian Veterans

**DOI:** 10.3390/ijerph21060796

**Published:** 2024-06-18

**Authors:** Ben Wadham, Lisa Andrewartha, Sharon Lawn, Ilke Onur, Laura Catherine Edney

**Affiliations:** 1 College of Education, Psychology and Social Work, Flinders University, Adelaide, SA 5000, Australia; ben.wadham@flinders.edu.au; 2 School of Education, La Trobe University, Melbourne, VIC 3000, Australia; l.andrewartha@latrobe.edu.au; 3 College of Medicine and Public Health, Flinders University, Adelaide, SA 5000, Australia; 4 College of Business, Government and Law, Flinders University, Adelaide, SA 5000, Australia; ilke.onur@flinders.edu.au; 5 Flinders University Institute of Mental Health and Wellbeing, Flinders University, Adelaide, SA 5000, Australia; laura.edney@flinders.edu.au

**Keywords:** veterans, mental health, wellbeing, military, social determinants, scoping review

## Abstract

Serving in the military can have significant impacts on the mental health of veterans and their families. Military personnel can be exposed to a range of physical stressors, psychological trauma, risky lifestyle factors, a regimented military culture, and inadequate support when transitioning out of service. This article reviews research on interventions designed to improve the mental health of Australian military veterans in order to synthesise current knowledge and identify gaps in the literature. Our scoping review followed PRISMA recommendations and comprised peer-reviewed literature published since 2000. The review demonstrates a dominance of psychologically driven research paradigms and interventions and a neglect of the importance of social factors in shaping veteran mental health. There is a wide range of interventions available; however, the literature is narrow and limited. We found little evidence that the lived experience of veterans had been harnessed in program design or delivery. We argue the need for a holistic approach that moves beyond psychological and biological understandings of health and wellbeing to incorporate social and cultural determinants. Future research could adopt a stronger multidisciplinary approach, increased socio-cultural understanding, and greater consideration of the lived experience of veterans and their families.

## 1. Introduction

Nearly half a million living Australians have formerly served in the Australian Defence Force (ADF) [1]. The Australian Department of Veterans’ Affairs (DVA) provides a broad definition of veteran as anyone who has served in uniform in the Australian Defence Force full time for at least one day [2]. Military service can have significant impacts on the mental health of veterans and their families [3,4].

Military service can affect physical, mental, and social wellbeing due to factors such as intense physical activity, exposure to physical and psychological trauma, and lifestyle factors, such as excessive alcohol consumption [5]. The military is an intensely social institution - esprit de corps and camaraderie are highly significant. When one serves, they are likely to develop a strong sense of military identity, purpose, and belonging (or community). Military culture can also be described as rigid, hierarchical, and regimented, with an emphasis on strength and stoicism [6]. Many military personnel do not utilise mental health services due to stigma around mental health issues and service use in the military [7]. Illness and injury can be viewed as weaknesses, contributing to an environment of harassment and abuse, surrounded by a code of silence, which together stifle help-seeking for mental health concerns and can have lasting health impacts [6].

ADF members are provided with structured healthcare while serving; however, this care often ceases once veterans transition out of the service, unless the veterans have been assessed as having ongoing healthcare needs relating to their military service. The stark difference between military and civilian life can be difficult to navigate. The shift from being part of a unified and structured environment in the military to being an individual in the civilian community can lead to what has been described as ‘reverse culture shock’ [8]. Challenges can include the loss of community, friendships, status, and purpose, as well as dealing with the impacts of service on psychological and physical health [9,10,11]. 

Research shows that specific social factors may have adverse impacts on veterans’ mental health, including social isolation and issues with interpersonal relationships [5,10]. Veterans can face difficulties in areas of employment, education, health, social integration, and identity [12,13]. Veterans are at increased risk of post-traumatic stress disorder (PTSD), depression, anxiety, and substance abuse [14]. Ex-serving men have a 24 percent higher suicide rate than the national average, and ex-serving women have a suicide rate of approximately twice the national average [1].

Considering that these difficulties relate to physical, mental, and social aspects of transition, there is a need to provide integrated care across physical, mental, and social issues and needs for veterans [5]. This approach is often understood as the biopsychosocial model of healthcare assessment and service provision. Engel [15] theorised the biopsychosocial model and described how the interconnection between biological, psychological, and social factors influenced mental health. The biopsychosocial model comprises three domains—the biological domain (e.g., age, biomechanics, comorbidity, gender, genetics, metabolic factors, neurochemistry, pathophysiology, and physical), the psychological domain (e.g., addictions, attitudes/beliefs, cognitive factors, developmental issues, expectations, literacy/health literacy, mental illness, past experiences, personality, preferences, psychological stress, readiness to change, self-efficacy, and self-esteem), and the social domain (e.g., economic factors, employment/occupation, environment/geography, ethnicity/culture/race, family/social support and relationships, health provider/system factors, housing, and language proficiency) [16].

We return to this biopsychosocial philosophy of care at a time when the Australian veteran health and welfare system is undergoing significant scrutiny and change. One clear critique has been that mental health, including self-harm and suicidality, have not been adequately addressed because the biomedical/legal model remains dominant at the expense of a more holistic, human, and compassionate paradigm. Borrell-Carrió et al. [17] explain how Engel’s early work was an attempt to challenge the dehumanisation of medicine and disempowerment of patients, and espouse a more compassionate stance:

*… [*Engel*] formulated his model at a time when science itself was evolving from an exclusively analytic, reductionistic, and specialized endeavour to become more contextual and cross-disciplinary. Engel did not deny that the mainstream of biomedical research had fostered important advances in medicine, but he criticized its excessively narrow (biomedical) focus for leading clinicians to regard patients as objects and for ignoring the possibility that the subjective experience of the patient was amenable to scientific study.*

(p. 576)

Further calls for reform to mental health policy and service delivery, more broadly, have been led particularly by the mental health lived experience advocacy community in Australia and internationally, with calls on mental health services to shift from a focus on individual deficits in functioning to a strengths-based recovery approach. This “individual-deficit model of diagnosis for mental health asserts that the symptoms of mental illness are the result of personal limitations” [18] (p. 447), leaving the social, environmental, and system contexts in which mental health challenges develop (such as trauma, abuse, and discrimination) unacknowledged and, therefore, unaddressed.

Compton and Shim [19] (p. 419) explain that social and environmental factors have a role in the prevention and development of mental health challenges, as well as their treatment and improvement. The social determinants of veteran mental health are well represented in the Department of Veterans’ Affairs (DVA) ‘Wheel of Wellbeing of Veterans and Their Families’. The Wheel outlines eight life domains, including education and skills, employment and meaningful activity, income and finance, recognition and respect, health, housing, social support, and connection, and justice and safety [20]. Compton and Shim [10] further explain that:

*… the widespread use of the biopsychosocial model in formulating both etiology and course/outcomes testifies to the broad recognition of the importance of both biological and social factors in shaping behavioral disorders … To effectively treat—and ultimately prevent—mental illnesses and substance use disorders (and promote mental health more generally), our field must carefully evaluate the role that nongenetic social and environmental factors play in bringing about poor mental health and in causing and worsening mental illnesses. In doing so, we must consider the roles of social justice, political will and power, policy action, resource distribution, and program development and implementation in addressing these factors.*

(p. 419)

There is widespread recognition of the high need for interventions to support veteran mental health. There is limited evidence, however, on the range of interventions offered to improve wellbeing for Australian veterans. In this review, we aimed to provide the first comprehensive overview of mental health interventions provided to Australian veterans reported in the academic literature. This included studies involving veterans who were currently or previously serving in the military at the time when the intervention was being researched. Our secondary aims were to detail the research paradigms defining the identified research, the intervention types evaluated, the factors targeted during interventions, and the use of lived experience in intervention development. 

## 2. Materials and Methods

This review followed the Preferred Reporting Items for Systematic Reviews and Meta-Analyses (PRISMA) recommendations [21]. A scoping review methodology was deemed the most appropriate method to address our aim to identify the breadth of evidence on mental health interventions for Australian veterans. A scoping review is able to synthesise the current knowledge and identify knowledge gaps in the area, highlighting implications for policy and practice. Formal quality appraisals were not undertaken, as the aim of a scoping review is to provide all evidence on the topic regardless of quality [22,23].

### 2.1. Search Strategy

A comprehensive literature search was conducted in peer-reviewed literature to identify relevant articles published between January 2000 and April 2022 in Medline, PsycINFO, CINAHL, EconLit, Embase, Cochrane Collaboration Library, Informit, and Scopus. The literature search included Medical Subject Headings (MeSH) and keyword searches to describe the target population (veterans) and location (Australia). The research team and a public health sciences librarian developed the search strategy (see Supplementary Table S1 for the Medline search strategy) using an iterative approach to ensure rigour and defensibility [22].

### 2.2. Inclusion Criteria

This systematic review included quantitative and qualitative studies conducted on Australian veterans currently or previously employed either full- or part-time in the Australian Defence Force. International samples and samples of family members were excluded. There were no exclusion criteria applied to the types of intervention. The inclusion criteria were that the intervention targeted mental health promotion or a mental health condition and was not a descriptive study of the predictors of mental health or perspectives on mental health. There were no exclusion criteria applied to the presence or type of comparator, and no restrictions on the measurement of outcomes. Articles were excluded if they were not peer-reviewed, not reported in English, published prior to 2000, or if they were a review study or an opinion or discussion publication.

### 2.3. Data Extraction

An extraction form was developed and tested to ensure all relevant information was extracted. Data were extracted by one reviewer (LA) and a sample of 10% was reviewed by each of the other authors (LCE, BW, SL, and IO). Information was extracted by population, intervention, comparator, and outcomes, and included bibliographic details, study design, setting, recruitment, allocation, eligibility criteria, characteristics of the sample (e.g., age, gender, length of service, and time since service), intervention details, including target group, context, and comparator, outcome measures, intervention costs, and key study findings relating to effectiveness and costs, where available.

## 3. Results

**Figure 1:**
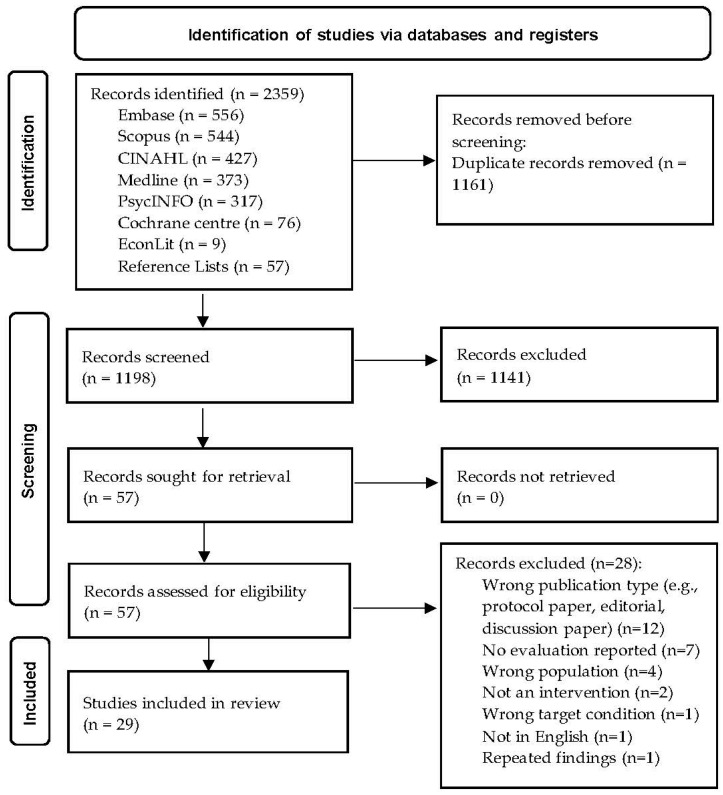
PRISMA flow diagram (from Page et al. [34]).

A total of 2359 records were identified in the peer-reviewed literature, reduced to 1198 after removing duplicates. Titles and abstracts were each screened by two authors against the inclusion and exclusion criteria, and any conflicts were resolved with discussion. A total of 57 records were included for full-text review, with 28 excluded due to being the wrong publication type, reported in a language other than English, or not reporting on the impact of the intervention on Australian veterans (see Figure 1 for further details). A total of 29 studies were included in the final review.

### 3.1. Study and Participant Characteristics

Study and participant characteristics are presented in Table 1. Approximately three-quarters of the articles (n = 22, 76%) were published from 2010 onwards. Small sample sizes were common, where approximately half of the studies (n = 16, 55%) had fewer than 100 participants in total, including both intervention and control groups, where applicable. A total of 7 studies had between 100 and 500 participants (n = 7, 24%), and the remaining 6 studies had over 1000 participants (n = 6, 21%). Convenience sampling methods were often used, where participants were recruited through either a specific location or service. Eleven studies (38%) comprised solely of male samples, eight (28%) had samples where men comprised at least three-quarters of the sample, six (21%) had a more even balance of males and females, and four studies (14%) did not specify gender. The conditions most commonly targeted were PTSD (targeted in n = 20 studies, 69%), depression (n = 21, 72%), anxiety (n = 18, 62%), problematic alcohol use (n = 15, 52%), and problematic anger (n = 10, 34%).

Most studies comprised formerly serving ADF members only; however, three studies included samples comprising both current and former ADF personnel [24,25,26], and one study included actively serving personnel only [27]. Three studies included veterans and their partners [28,29,30] and two studies included veterans and widow(er)s [31,32]. Where information on service or deployment experience was available (n = 12, 41%), the most common deployment was to Vietnam. Only two studies specifically recorded the time since military service in number of years [30] or months [26], and less than one-third of the studies (28%) included information on the participants’ length of service.

A variety of study designs was employed. Just over one-quarter of studies (n = 8, 28%) reported on randomised controlled trials (RCTs). In these studies, participants were randomly allocated to an intervention condition or a control group of usual care or delayed intervention. Participants were usually unable to be blinded to the condition, although one study provided placebo attention training to blind conditions [26].

Almost three-quarters of the studies (n = 21, 72%) were not RCTs and instead employed alternative and less rigorous designs. The non-RCT studies included examinations of existing treatment datasets, clinical case series, and quasi-experimental observational designs. Four of the non-RCT studies included comparison groups, which were typically different intervention conditions. In one study, comparisons were made between two groups of participants—Vietnam veterans and peacekeeper veterans—who received the same form of PTSD treatment [33]. Much of the research was supported by funding from the Australian DVA. Approximately three-quarters of the articles (n = 22, 76%) acknowledged funding support from the Australian DVA and/or were led by researchers from Phoenix Australia, which is partially funded by the DVA.

**Table 1:** Study and participant characteristics.

Lead Author (Year)	Participant Type	Overall Participants (n)	Gender	Age Mean ± Standard Deviation (M±)	Targeted Conditions	Outcome Measures
Allen et al. (2011) [31]	Veterans, war widows(ers), informal carers	97	50.5% male	M 83 ± 6 years	Depression, anxiety, stress, alcohol use, quality of life.	DASS21, AUDIT, WHOQoL-BREF, evaluation survey.
Battersby et al. (2013) [35]	Vietnam veterans	77	NR	Intervention: M 60.55 ± 3.40 years Usual care: M 60.18 ± 2.24 years	PTSD, alcohol use, quality of life, anxiety, depression, anger, dyadic adjustment, self-care habits.	AUDIT, AQoL, HADS, PCL-M, DAR, ADAS, PIH, P&G.
Beattie et al. (2013) [28]	Vietnam veterans and partners	43	58% male	NR	Alcohol use, quality of life, anxiety, depression, anger, dyadic adjustment.	AUDIT, AQoL, HADS, DAR, ADAS.
Bird (2015) [24]	Current and ex-serving ADF members, and 1 ex-emergency services worker	20	Male	M 43.3 ± 11.3 years. Range 31–66 years	Depression, anxiety, stress, positive and negative interactions, general perceived self-efficacy, life satisfaction.	DASS 21, PNI, GSE, LSQ.
Byles et al. (2004) [32]	Veterans and war widows	1569	NR	NR	Medical outcomes, healthcare use, admission to hospital, death.	SF-36, general items assessing healthcare use, including admission to hospital in the previous year. Participants cross-checked against the National Death Index after completion of all interviews.
Carter et al. (2013) [36]	Vietnam veterans	25	Male	Intervention: M 58.5 ± 3.8 years Delayed: M 58.4 ± 4.8 years	PTSD, depression, quality of life, alcohol use.	CAPS, PCL-M, CES-D, WHOQoL, AUDIT.
Cash et al. (2018) [27]	Actively serving ADF members	12	Male	M 36.3 ± 6.1 years. Range 27–48 years	PTSD, anxiety, depression, alcohol use, anger.	CAPS-5, DAR-5, STAXI-2, PCL-5), HADS.
Creamer et al. (2006) [37]	Vietnam veterans	2223	Male	M 52.29 ± 5.05 years	PTSD, combat exposure, alcohol use, general health, anxiety, depression, general functioning, anger.	CES, PCL, AUDIT, GHQ-28, HADS, FAD, SF-12.
Creamer et al. (2002) [38]	Vietnam veterans	202	Male	Inpatient–outpatient: M 51.2 ± 4.7 years Day hospital: M 52.3 ± 5.3 years	PTSD, alcohol use, general health, anxiety, depression, general functioning, anger.	CAPS, PCL, AUDIT, GHQ-28, HADS, FAD, SF-12.
Dell et al. (2022) [25]	Current and ex-serving ADF members	134	88% male	Massed prolonged exposure: M 44.29 ± 10.83 years Standard prolonged exposure: M 46.69 ± 12.68 years	PTSD, anxiety, depression, anger, quality of life, disability, alcohol use.	CAPS-5, PCL, HADS, DAR-5, AQoL, WHODAS 2.0, AUDIT.
Forbes et al. (2005) [33]	Vietnam veterans and peacekeeper veterans	129	NR	Veterans: M 52.69 ± 3.02 years Peacekeepers: M 35.68 ± 7.05 years	PTSD, anxiety, depression, alcohol use, anger.	CAPS, PCL, HADS, AUDIT, anger items of the War Stress Inventory used by the US VA.
Forbes et al. (2013) [39]	Veterans (92% served in Vietnam)	1548	Male	M 54.9 ± 8.68 years	PTSD, combat exposure, anxiety, depression, alcohol use.	CAPS, CES, PCL, HADS, AUDIT.
Forbes et al. (2008) [40]	Veterans	4339	Male	M 54.40 ± 8.86 years	PTSD, anxiety, depression, alcohol use.	CAPS, PCL, HADS, AUDIT.
Forbes et al. (2003) [41]	Vietnam veterans	12	Male	Range 45–50 years	PTSD, target nightmare frequency, target nightmare intensity, general nightmare frequency, general nightmare intensity, distress, depression, anxiety.	Target nightmare frequency, target nightmare intensity, general nightmare frequency, general nightmare intensity, IES, BDI, BAI, GSI SCL.
Khoo et al. (2011) [42]	Veterans (68% served in Vietnam)	496	99.8% male	M 53 ± NR years. Range 25–74 years	PTSD, dyadic adjustment, alcohol use, anger, anxiety, depression, quality of life.	PCL, ADAS, AUDIT, DAR, HADS, WHOQOL-BREF.
Lloyd et al. (2015) [43]	Veterans	100	82% male	M 43.82 ± 14.59 years	PTSD.	PCL.
Lloyd et al. (2014) [44]	Veterans (66% served in Vietnam)	59	Male	NR	PTSD, depression, anxiety, anger, alcohol use.	CAPS, BDI, STAI, DAR, AUDIT.
Metcalf et al. (2022) [26]	ADF members who were leaving within the next 4 months or had left within the past 4 months	59	81% male	Range 18 to ≥55 years	PTSD, work and social adjustment.	PCL-5, WASAS.
Nursey et al. (2020) [45]	Veterans	8	87.5% male	M 37.52 ± 6.93 years	PTSD, depression.	CAPS-5, HAM-D.
O’Donnell et al. (2013) [29]	Veterans, partners, and families	312	54% male	M 51.29 ± 14.63 years	Depression, anxiety, stress, alcohol use	DASS-21, AUDIT.
Otter et al. (2004) [46]	Vietnam veterans	14	Male	M 55 years	Work and lifestyle, motivation, anger levels and psychological changes, daily habits, resilience and energy levels, and social support.	Three focus groups.
Phelps et al. (2018) [47]	Veterans (66% served in Vietnam)	2685	98.8% male	M 55.92 ± 10.54 years	PTSD, alcohol use, anxiety, depression.	PCL, AUDIT, HADS.
Ray et al. (2010) [48]	Veterans	9	Male	Range 56–75 years	PTSD, mental health, social functioning, interpersonal problems, depression, distress.	CIDI, HoNOS, GAF, SO-EAS, IIP-32, IES-R, BDI-II, SUDS.
Romaniuk et al. (2018) [30]	Veterans and partners	47	64% male	Individuals: M 50.28 ± 14.59 years Range 26–72 years Couples: M 42.12 ± 10.03 years Range 29–68 years	PTSD, depression, anxiety, stress, happiness, quality of life, enjoyment, and satisfaction.	PCL-5, DASS-21, OHQ, Q-LES-Q-SF.
Romaniuk et al. (2019) [49]	Veterans	29	75.9% male	M 42.28 ± 9.67 years	PTSD, depression, anxiety, stress, happiness.	DASS-21, PCL-5, OHQ.
Roughead et al. (2013) [50]	Veterans, GPs, pharmacists	12 interventions with an average target of 33,000 veterans, 10,000 GPs, and 8500 pharmacists per intervention	NR	The target group was elderly veterans, but age range varied between interventions	Health claims related to the use of medications, including antidepressants and antipsychotics.	Evaluated using DVA administrative health claims data.
Shakespeare-Finch et al. (2020) [51]	Serving and ex-serving ADF members	53	91% male	Workshops: Median 62 ± 12.4 years. Range 35–79 years Telephone interviews: Average 55.8 ± 12.7. Range 31–80 years.	Experience using the PTSD Coach Australia mobile app.	MARS, uMARS, Qualitative data from focus groups at workshops and telephone interviews.
Watt et al. (2021) [52]	Veterans	37	62% male	Range <30 to >50 years	Sense of purpose, meaning, achievement, enjoyment, flow, belonging, and positive interaction.	Short self-report survey with questions about perceived benefits, current activities, and future engagement.
Wootton et al. (2010) [53]	Veterans	481	49% male	Intervention: M 78.5 ± 9 years Usual care: M 78.1 ± 10 years	Quality of life, medical costs.	SF-12, EQ-5D. Total medical costs from DVA records.

ADF, Australian Defence Force; GP, general practitioner; NR, not reported; PTSD, post-traumatic stress disorder.

### 3.2. Interventions

Intervention characteristics are presented in Table 2. Not all studies provided detail about which specific intervention modalities had been employed. While there was a wide range of intervention types, their overall intent was focused on addressing individuals’ mental ill-health within a ‘treatment’ focus, with little or no evidence of targeting or measuring impacts on wider social determinants of mental health. It was relatively common for interventions to incorporate some form of cognitive behavioural therapy (CBT). Specific interventions included a mental health screening and referral pathway, the multi-component Flinders Program of chronic condition self-management care planning, the Stanford Program of group-based chronic disease self-management education, peer outdoor support therapy, home-based health assessments, prolonged exposure therapy, imagery rehearsal therapy, cognitive processing therapy, attention-control training, theta burst stimulation, an aerobic exercise program, equine-assisted therapy, a PTSD coaching app, a medicines advice and therapeutics education program, an online peer-delivered peer support program, an art-based program, and a yoga-based program. 

### 3.3. Lived Experience

Only six studies (21%) made reference to incorporating the lived experience of veterans in program design or delivery and harnessed their first-hand knowledge and experiences. One study used an online peer-delivered peer support program that was both developed and facilitated by a veteran [53], two studies included veterans as program facilitators to support program credibility [24,28], and another study included veterans as guest speakers [42]. Most articles did not include comprehensive detail about program development. Roughead et al. [50], however, reported that the MATES program involved ongoing consultation with veteran reference groups, and Shakespeare-Finch et al. [51] noted that the original version of the PTSD Coach app was developed in the United States in collaboration with veterans who had PTSD.

### 3.4. Outcomes

Methods typically involved administering a battery of psychological self-report questionnaires (see Table 1 for outcome measures and Supplementary Table S2 for the glossary of outcome measures). The most common methodology involved administering pre- and post-intervention questionnaires to measure changes in mental health symptoms. There was only one study that did not employ any surveys as part of the evaluation [46].

Participants were typically assessed using multiple instruments that had strong psychometric properties. PTSD symptoms were most commonly assessed using a version of the self-report PTSD Checklist (PCL; n = 14, 48%) and/or the Clinician-Administered PTSD Scale (CAPS; n = 9, 31%). Other frequently used self-report measures included the Alcohol Use Disorders Identification Test (AUDIT; n = 14, 48%), the Hospital Anxiety and Depression Scale (HADS; n = 11, 38%), the Dimension of Anger Reactions (DAR; n = 6, 21%), and the Depression Anxiety Stress Scale (DASS-21; n = 5, 17%). Generic quality of life scales, such as the Assessment of Quality of Life (AQoL) instrument or the World Health Organization Quality of Life (WHOQOL) instrument, were employed in eight of the studies (28%).

These methods and measures reflected a focus on the individual through the biopsychological lens of symptom improvement, without consideration of social or environmental contexts. Indeed, consideration of social factors was typically limited to the inclusion of psychosocial scales designed to assess the quality of interpersonal relationships. Eight studies (28%) included at least one psychosocial scale, specifically the Abbreviated Dyadic Adjustment Scale (ADAS), the General Functioning subscale of the Family Assessment Device (FAD), the Positive and Negative Interactions Scale (PNI), the Work and Social Adjustment Scale (WASAS), the Social Functioning Assessment Scale (SO-EAS), and/or the Inventory of Interpersonal Problems (IIP). None of the studies in this review looked more broadly at measuring social factors that can impact mental health and wellbeing, such as income, education, or housing, or at the socio-cultural aspects of service, separation, and military-to-civil transition.

Follow-up assessments were commonly conducted. Most studies (n = 24, 83%) incorporated post-intervention follow-up assessments to determine whether any improvements to mental health were maintained over time. Follow-up periods varied between studies, with the most common periods being 3 months, 6 months, 9 months, and/or 12 months after intervention.

One study by Byles et al. [32] was notable for reporting on an RCT with a relatively large sample size. It examined the effect of home-based health assessments over 3 years with 1569 community-living veterans and war widows. The researchers found that health assessments for older people may have small positive effects on quality of life for those who remain as residents in the community and may increase the probability of nursing-home placement, but do not make a significant difference to the probability of hospital admission or death.

Three RCTs indicated promising results in terms of reducing PTSD symptoms using different interventions. These interventions were mass prolonged exposure therapy [25], attention control training [26], and a yoga-based stress reduction program [36]. Other RCTs demonstrated the potential effectiveness of a chronic disease self-management care-planning approach (the Flinders Program) in reducing alcohol dependence [35] and the Stanford Program in providing self-management education to help veterans to manage comorbid alcohol and mental health conditions [28].

Almost all the non-RCTs showed promising results in terms of the participants’ self-reported improvements in mental health and quality of life following intervention. Five of the non-RCTs explicitly described the use of cognitive behavioural therapy (CBT) to treat PTSD symptoms in veterans. CBT was primarily delivered in group-based sessions; however, the length and setting varied between treatment programs. Statistically significant and sustained improvements in PTSD symptoms were reported after CBT, which is consistent with previous research [54,55,56,57]. On the basis of these non-RCTs alone, however, causality cannot be inferred regarding CBT and reduced PTSD symptoms.

One non-RCT study, by Roughead et al. [50], was noteworthy for including a uniquely large sample size. It examined changes in medicine use after participation in the Veterans’ Medicines Advice and Therapeutics Education Services (MATES) program. Medications targeting mental health included antidepressants and antipsychotics. Here, 12 specific interventions were examined with an average target of 33,000 veterans, 10,000 GPs, and 8500 pharmacists per intervention. Using DVA administrative health claims data, all programs that aimed to increase medicine use were found to be effective; however, mixed results were seen with programs that aimed to reduce inappropriate medicine use.

## 4. Discussion

This review represented a comprehensive examination of the academic literature on interventions designed to improve the mental health of Australian military veterans. While self-reported improvements in mental health and quality of life were reported across a range of interventions, the literature was limited in size and narrow in scope. The research and interventions appeared skewed towards psychiatric models of care, where the predominant paradigm was biological and psychological (or psychosocial), and the social determinants of mental health were largely overlooked. This review demonstrates that research in Australia on the mental health and illness of veterans is predominantly conducted in an objectivist paradigm, utilising positivist research methods, including randomised controlled trial designs. 

Few studies focussed on the social and cultural aspects of service, separation, and military-to-civil transition. As such, the ‘social’ appeared to be missing in the biopsychosocial understanding of veteran mental health and wellbeing, and a more holistic understanding of veterans’ needs and experiences was overlooked. This approach does not adequately recognise the influence of military systems and cultures on veteran mental health. Recent work by Wadham et al. [6] demonstrated how ADF systems and culture can lead to self-harm and suicidality. If these matters are not understood, then Australian interventions, and the national current and future policy environment that is determined and underpinned by the evidence arising from the existing research, are addressing issues that will continue to occur within more narrow understandings of veteran mental health and wellbeing that exist within current institutional settings. 

Importantly, consideration needs to be given to both the internal world of the person and the external world in which they live [58,59]. The Wheel of Wellbeing of Veterans and Their Families [20] captures the way mental health is influenced by education and skills, employment and meaningful activity, income and finance, recognition and respect, health, housing, social support, and connection, and justice and safety. As such, the ‘problem’ is not viewed as only residing within the person (i.e., seeing people for their deficits in need of correction by experts) but also residing within the wider environment acting upon the person (i.e., seeing people as having potential for growth if the environmental conditions in which they sit are supportive and enabling) [60]. We argue that this research, and the health and wellbeing of veterans, would be enhanced by bringing the social back into the biopsychosocial, and recognising the social as a distinct influence itself, not simply an addendum to the biological and psychological understandings and explanations of mental health.

This means attending to the biological and psychosocial as three distinct elements—the biological, the psychological, and the social. The social here must draw upon sociological and anthropological expressions of the human condition as well as existing psychosocial understandings. This also means focussing upon the regimes of living across the life course. This is achieved by assessing the manner in which the subject navigates the life domains of social determinants—health, housing, education, community, or recognition and respect, for example. Rose et al. [61] described these as ‘niches’, which are not fixed ‘environments’ but rather like the Bordieuan habitus, which is, “the way society becomes deposited in persons in the form of lasting dispositions, or trained capacities and structured propensities to think, feel and act in determinant ways, which then guide them” [62] (p. 316).

Few studies adopted somatic models of intervention and peer-based models of support based on mutuality, reciprocity, and other principles that underpin personal recovery. There was little evidence of the lived experience of veterans in program design or delivery. To create a more holistic picture of veteran mental health, future research could look to additional frameworks, such as the CHIME approach, which considers Connectedness, Hope, Identity, Meaning, and Empowerment [63], and Keyes’ two-continuum model of mental health, which comprises two distinct dimensions—the presence or absence of mental health and the presence or absence of mental illness [64]. The use of alternative scales could also be valuable, for example, the Self-Efficacy for Personal Recovery Scale [65], the Recovery Assessment Scale (RAS-DS) [66], and the Warwick-Edinburgh Mental Well-Being Scale [67].

## 5. Conclusions

This review found that the available academic literature on interventions to improve the mental health of Australian veterans is limited in scope. The quality of veteran mental health research in Australia would be improved by a stronger multidisciplinary approach, a growth of the socio-cultural understanding of veteran and family wellbeing, and subject to this, a shift to include the lived experience of veterans in the design of research and interventions to meet their needs. Additional evaluations of promising interventions are warranted to inform more holistic evidence-based practice that also encompasses the social determinants of health and wellbeing and can better support veterans during and after their service.

## Figures and Tables

**Table 2:** Intervention characteristics.

Lead Author (Year)	Purpose	Study Design	Setting	Intervention	Follow-Up Period
Allen et al. (2011) [31]	Development and evaluation of a mental health screening and referral pathway	Pre- and post-intervention surveys	Community nursing services in urban and rural/semi-rural areas	The pathway included screening tools to support nurses’ decision-making regarding referral options.	Variable (up to 42 days)
Battersby et al. (2013) [35]	Evaluation of the Flinders Program for veterans	Waitlist RCT	Metropolitan community, Adelaide	The Flinders Program aimed to engage veterans in their own care by providing a structured clinical process for health professionals. Control: usual care.	9 months and 18 months
Beattie et al. (2013) [28]	Evaluation of self-management education for veterans with comorbid alcohol and mental health conditions	Part of an RCT	NR	The 6-week Stanford Program taught problem-solving and decision-making skills to activate healthful behaviours. Control: usual care.	9 months
Bird (2015) [24]	Evaluation of a peer outdoor support therapy program	Pre- and post-intervention surveys	Outdoors, remote South Australia	The peer outdoor support therapy (POST) program included a 6-day camp, approximately 14 structured CBT-based group sessions, skill building activities, informal gatherings, and one-on-one debriefings.	6 days and 2 months
Byles et al. (2004) [32]	Examination of the effect of home-based health assessments over 3 years	RCT	Communities in 10 geographic regions of New South Wales and Queensland	Home-based health assessments: Group 1: annual visits, with a report to the GP, and telephone follow-up after each visit. Group 2: as Group 1, with a second report to the GP after telephone follow-up. Group 3: 6-monthly visits, with a report to the GP, and telephone follow-up after each visit. Group 4: as Group 3, with a second report to the GP after each telephone follow-up. Control: usual care.	1 year, 2 years, and 3 years
Carter et al. (2013) [36]	Examination of a yoga-based stress reduction program for PTSD	RCT	Brisbane	The Sudarshan Kriya Yoga (SKY) intervention was adapted for veterans and consisted of 22 h of guided yoga instruction over a period of 5 days. Control: delayed intervention.	6 weeks and 6 months post-intervention
Cash et al. (2018) [27]	Examination of the effectiveness of a CBT-based anger intervention for PTSD	Clinical case series	Clinical	Participants received 12 sessions of CBT developed to target anger in the context of military PTSD.	None. Pre-treatment and post-treatment assessment only
Creamer et al. (2006) [37]	Examination of PTSD treatment outcomes involving a predominately CBT-based approach	Naturalist study comprising consecutive treatment admissions to examine outcomes two years following treatment.	Clinical	The study examined 19 different PTSD programs across Australia, including inpatient–outpatient models, day hospital models, and low-intensity outpatient models. Treatment predominantly used a group-based CBT approach.	6 months, 12 months, and 24 months post-admission
Creamer et al. (2002) [38]	Comparison of treatment outcomes of inpatient–outpatient programs and day hospital programs for PTSD	Quasi-experimental observational design	Clinical	Inpatient–outpatient treatment programs for PTSD were compared with day hospital programs.	3 months and 9 months post-discharge
Dell et al. (2022) [22]	Examination of the effect of massed versus standard prolonged exposure therapy on PTSD	Single-blinded multi-site non-inferiority RCT	Clinical health clinics across Australia and via telehealth during COVID-19	Massed prolonged exposure (MPE) therapy was delivered rapidly over 2 weeks and compared with standard 10-week prolonged exposure (SPE) therapy.	4 weeks and 12 weeks post-commencement. 12-month data collection is ongoing.
Forbes et al. (2005) [33]	Examination of treatment outcomes for Vietnam veterans and peacekeeper veterans with PTSD	Examination of intake and treatment outcome data	Clinical	PTSD treatment programs.	3 months post-treatment
Forbes et al. (2013) [39]	Examination of impact of combat and non-military trauma on symptom reduction following primarily CBT-based treatment for PTSD	Examination of intake and treatment outcome data	Clinical	The specialist veteran PTSD program was group-based, primarily CBT in orientation, and delivered over 12 weeks.	9 months post-treatment
Forbes et al. (2008) [40]	Evaluation of five group-based CBT models for veterans with combat-related PTSD	Examination of consecutive treatment admissions	Five CBT-based program models with different levels of intensity and settings.	CBT-based program models: 1. High-intensity inpatient–outpatient programs. 2. High-intensity residential programs. 3. Moderate-intensity day hospital programs. 4. Moderate-intensity regional day hospital programs. 5. Low-intensity programs.	3 months and 9 months post-discharge
Forbes et al. (2003) [41]	Examination of Imagery Rehearsal in the treatment of PTSD	Examination of 12-month follow-up data of a pilot study	NR	Imagery Rehearsal Therapy was designed to reduce nightmare frequency and intensity and was delivered as 6 once weekly, 90 min group sessions.	3 months and 12 months
Khoo et al. (2011) [42]	Evaluation of group CBT for service-related PTSD	Pre- and post-intervention surveys	Private psychiatric hospital in Queensland	The CBT program was delivered via a manual.	3 months and 9 months post-treatment
Lloyd et al. (2015) [43]	Examination of treatment outcomes of cognitive processing therapy (CPT) for PTSD	Examination of treatment outcomes	A community-based mental health service in Australia, the Veterans and Veterans Families Counselling Service (VVCS)	Cognitive processing therapy (CPT) sessions.	None. Pre-treatment and post-treatment assessment only
Lloyd et al. (2014) [44]	Examination of treatment outcomes of cognitive processing therapy (CPT) for PTSD	RCT	Three community-based Veterans and Veterans Families Counselling Service (VVCS) offices in Australia	Cognitive processing therapy (CPT) was provided as a 12-session treatment for PTSD and delivered according to a manual. Individual 60 min therapy sessions were delivered twice a week. Control: usual care.	3 months post-treatment
Metcalf et al. (2022) [26]	Examination of the impact of attention-control training on PTSD symptoms and functional impairment	Two-arm parallel-group RCT	Participants were treatment-seeking military personnel and veterans recruited from Open Arms–Veteran and Families Counselling centres	Participants received attention-control training, where they were presented with both neutral (e.g., “chair”) and threat-related (e.g., “death”) words and asked to respond to a target cue. Control: placebo task.	None. Pre-treatment and post-treatment assessment only
Nursey et al. (2020) [45]	Exploration of theta burst stimulation for PTSD	Case series, repeated-measures design	Participants received treatment sessions at the Monash Alfred Psychiatry Research Centre in Melbourne	20 bilateral intermittent theta burst stimulation (iTBS) treatments were delivered as 1 session per day, 5 days per week, over a 4-week period.	3 months
O’Donnell et al. (2013) [29]	Examination of the effect of centre-based counselling	Single group design	Centre-based counselling was provided through the Veterans and Veterans Families Counselling Service (VVCS)	Centre-based counselling.	12 months post-commencement
Otter et al. (2004) [46]	Examination of the experiences of an exercise program	Qualitative exploratory study using a grounded theory approach. This approach used a systematic set of procedures to develop an inductively derived theory.	A community exercise rehabilitation program	The 40-week supervised aerobic exercise class program, which specifically catered to Vietnam veterans, consisted of low- to moderate-intensity exercise to music.	None
Phelps et al. (2018) [47]	Investigation of the patterns of change in symptom clusters following standard treatment for PTSD	Examination of data collected as part of routine program participation	Clinical	Participants were involved in accredited group treatment programs. These programs incorporated 20 to 30 treatment days over a 3-month period. During this time, 6 to 10 participants per group received a combination of individual and group therapy.	3 months and 9 months post-discharge
Ray et al. (2010) [48]	Examination of group-based interpersonal psychotherapy for veterans with PTSD	Pre- and post-surveys of intervention and waitlist group	Outpatient program	An outpatient group-based interpersonal psychotherapy program for PTSD.	2 months and 4 months
Romaniuk et al. (2018) [30]	Evaluation of equine-assisted therapy for veterans who identify as ‘wounded, injured, or ill’	Non-controlled within-subjects longitudinal design and between-subjects analysis	A live-in residential equine-assisted therapy program	An individual program of equine-assisted therapy for veterans was compared with a couples’ program.	3 months post-intervention
Romaniuk et al. (2019) [49]	Evaluation of an online program for veterans with PTSD	Non-controlled, within-subject, longitudinal design	An online program	The ‘Post-War: Survive to Thrive Program’ was an online, peer developed and delivered program. The program was designed to assist with the management of commonly occurring mental health symptoms among veterans.	3 months and 6 months post-commencement
Roughead et al. (2013) [50]	Examination of changes in medicine use after the MATES health promotion program	Evaluated using DVA administrative health claims data	General practice	The Veterans’ Medicines Advice and Therapeutics Education Services (MATES) program aimed to improve medication use and health outcomes by delivering interventions to general practitioners, pharmacists, and veterans.	20 months post-intervention
Shakespeare-Finch et al. (2020) [51]	Evaluation of an app for people with PTSD and trauma-related symptoms	Evaluation using quantitative and qualitative data on user experience. Qualitative data were analysed using reflexive thematic analysis.	Use of an app	Participants used the PSTD Coach Australia app.	None
Watt et al. (2021) [52]	Evaluation of the Arts for Recovery, Resilience, Teamwork, and Skills (ARRTS) program	Third evaluation of the program using survey data	NR	The program comprised art-based activities.	3 months and 6 months post-program
Wootton et al. (2010) [53]	Examination of telephone-supported care coordination	RCT	General practice in Brisbane	Participants received care coordination that addressed their individual needs and access to a range of resources, including telephone counselling and patient support. Control: usual care.	12 months

CBT, cognitive behavioural therapy; DVA, Department of Veterans’ Affairs; GP, general practitioner; NR, not reported; PTSD, post-traumatic stress disorder; RCT, randomised controlled trial.

## Data Availability

All available data have been reported in the manuscript.

## Supplementary Materials

The following supporting information can be downloaded at: https://www.mdpi.com/article/10.3390/ijerph21060796/s1. Table S1: Medline search strategy. Table S2: Glossary of outcome measures.
