# Supplementary material for: A Scoping Review of Interventions Targeting the Mental Health of Australian Veterans
Source: Int J Environ Res Public Health. 2024 Jun 18;21(6):796. doi: 10.3390/ijerph21060796 (PMC11204173; doi:10.3390/ijerph21060796)
Supplement: Supplementary file 1 [file ijerph-21-00796-s001.zip › ijerph-3033166-supplementary.pdf]

Supplementary File - Table S1: Medline Search Strategy

**Ovid MEDLINE(R)**	
**1**	Veterans/
**2**	(Veteran or veterans or ex-serving or ex-defense* or Ex-service* or ex-militar* or past master* or past-master*).tw,kf.
**3**	((retired or retire or ex-member* or reintegrat* or transition*) adj5 (militar* or navy or army or navy or sailor* or solider* or enlisted or combat or adf or officer* or air force or air-force or defense or reservist* or reserves or trouper* or medical corp* or war or wars)).tw,kf.
**4**	or/1-3
**5**	Retirement/
**6**	Military Personnel/ or military deployment/ or exp warfare/
**7**	5 and 6
**8**	4 or 7
**9**	exp Australia/
**10**	(australia* or "new south wales" or sydney or queensland* or Brisbane or hobart or tasmania or northern territory or darwin or canberra or aborig* or "torres strait" or victoria or melbourne or adelaide or perth or woodside or Bungendore or Orchard Hills or Denman or Berrimah or Pine Gap or Port Philip or duntroon or Holsworthy or Adamstown or Mount Bundey or Berry Springs or Timber Creek or Cabarlah or Canungra or Enoggera or Townsville or Oakey or darling downs or Shoalwater Bay or cairns or Cowley Beach or Glenorchy or Launceston or Devonport or Warrane or Richmond or Burnie or Albury or Wodonga or Puckapunya or Swanbourne or Karrakatta or Lancelin or Bindoon or Karratha or wagga or Newcastle or Katherine or Weipa or exmouth or garden island).mp.
**11**	or/9-10
**12**	8 and 11
**13**	limit 12 to yr="2010 -Current"

**APA PsycInfo**	
**1**	exp Military Veterans/
**2**	(Veteran or veterans or ex-serving or ex-defense* or Ex-service* or ex-militar* or past master* or past-master*).ti,ab,id.
**3**	((retired or retire or ex-member* or reintegrat* or transition*) adj5 (militar* or navy or army or navy or sailor* or solider* or enlisted or combat or adf or officer* or air force or air-force or defense or reservist* or reserves or trouper* or medical corp* or war or wars)).ti,ab,id.
**4**	or/1-3
**5**	military personnel/ or air force personnel/ or army personnel/ or commissioned officers/ or enlisted military personnel/ or marine personnel/ or military attrition/ or military deployment/ or military duty status/ or military enlistment/ or military medical personnel/ or military psychologists/ or navy personnel/ or rotc students/ or volunteer military personnel/ or combat experience/ or military families/ or military psychiatry/ or war/ or nuclear war/
**6**	reintegration/ or retirement/
**7**	5 and 6
**8**	4 or 7
**9**	(australia* or "new south wales" or sydney or queensland* or Brisbane or hobart or tasmania or northern territory or darwin or canberra or aborig* or "torres strait" or victoria or melbourne or adelaide or perth or woodside or Bungendore or Orchard Hills or Denman or Berrimah or Pine Gap or Port Philip or duntroon or Holsworthy or Adamstown or Mount Bundey or Berry Springs or Timber Creek or Cabarlah or Canungra or Enoggera or Townsville or Oakey or darling downs or Shoalwater Bay or cairns or Cowley Beach or Glenorchy or Launceston or Devonport or Warrane or Richmond or Burnie or Albury or Wodonga or Puckapunya or Swanbourne or Karrakatta or Lancelin or Bindoon or Karratha or wagga or Newcastle or Katherine or Weipa or exmouth or garden island).mp.
**10**	8 and 9
**11**	limit 10 to yr="2000 -Current"

Embase	
**1**	veteran/
**2**	(Veteran or veterans or ex-serving or ex-defense* or Ex-service* or ex-militar* or past master* or past-master*).tw,kf.
**3**	((retired or retire or ex-member* or reintegrat* or transition*) adj5 (militar* or navy or army or navy or sailor* or solider* or enlisted or combat or adf or officer* or air force or air-force or defense or reservist* or reserves or trouper* or medical corp* or war or wars)).tw,kf.
**4**	or/1-3
**5**	military phenomena/ or air force/ or army/ or military deployment/ or military research/ or military service/ or navy/ or terrorism/ or war/ or warfare/ or military personnel/
**6**	community reintegration/ or retirement/
**7**	5 and 6
**8**	4 or 7
**9**	exp Australia/
**10**	(australia* or "new south wales" or sydney or queensland* or Brisbane or hobart or tasmania or northern territory or darwin or canberra or aborig* or "torres strait" or victoria or melbourne or adelaide or perth or woodside or Bungendore or Orchard Hills or Denman or Berrimah or Pine Gap or Port Philip or duntroon or Holsworthy or Adamstown or Mount Bundey or Berry Springs or Timber Creek or Cabarlah or Canungra or Enoggera or Townsville or Oakey or darling downs or Shoalwater Bay or cairns or Cowley Beach or Glenorchy or Launceston or Devonport or Warrane or Richmond or Burnie or Albury or Wodonga or Puckapunya or Swanbourne or Karrakatta or Lancelin or Bindoon or Karratha or wagga or Newcastle or Katherine or Weipa or exmouth or garden island).mp.
**11**	or/9-10
**12**	8 and 11
**13**	limit 12 to yr="2010 -Current"

Econlit	
**1**	(Veteran or veterans or ex-serving or ex-defense* or Ex-service* or ex-militar* or past master* or past-master*).ti,ab,kw.
**2**	((retired or retire or ex-member* or reintegrat* or transition*) adj5 (militar* or navy or army or navy or sailor* or solider* or enlisted or combat or adf or officer* or air force or air-force or defense or reservist* or reserves or trouper* or medical corp* or war or wars)).ti,ab,kw.
**3**	(J26 and D74).sh.
**4**	or/1-3
**5**	O56.sh.
**6**	(australia* or "new south wales" or sydney or queensland* or Brisbane or hobart or tasmania or northern territory or darwin or canberra or aborig* or "torres strait" or victoria or melbourne or adelaide or perth or woodside or Bungendore or Orchard Hills or Denman or Berrimah or Pine Gap or Port Philip or duntroon or Holsworthy or Adamstown or Mount Bundey or Berry Springs or Timber Creek or Cabarlah or Canungra or Enoggera or Townsville or Oakey or darling downs or Shoalwater Bay or cairns or Cowley Beach or Glenorchy or Launceston or Devonport or Warrane or Richmond or Burnie or Albury or Wodonga or Puckapunya or Swanbourne or Karrakatta or Lancelin or Bindoon or Karratha or wagga or Newcastle or Katherine or Weipa or exmouth or garden island).mp.
**7**	or/5-6
**8**	4 and 7

CINAHL	
**S13**	S8 AND S11
**S12**	S8 AND S11
**S11**	S9 OR S10
**S10**	TI ((australia* OR "new south wales" OR sydney OR queensland* OR Brisbane OR hobart OR tasmania OR "northern territory" OR darwin OR canberra OR aborig* OR "torres strait" OR victoria OR melbourne OR adelaide OR perth OR woodside OR Bungendore OR "Orchard Hills" OR Denman OR Berrimah OR "Pine Gap" OR "Port Philip" OR duntroon OR Holsworthy OR Adamstown OR "Mount Bundey" OR "Berry Springs" OR "Timber Creek" OR Cabarlah OR Canungra OR Enoggera OR Townsville OR Oakey OR "darling downs" OR "Shoalwater Bay" OR cairns OR "Cowley Beach" OR Glenorchy OR Launceston OR Devonport OR Warrane OR Richmond OR Burnie OR Albury OR Wodonga OR Puckapunya OR Swanbourne OR Karrakatta OR Lancelin OR Bindoon OR Karratha OR wagga OR Newcastle OR Katherine OR Weipa OR exmouth OR "garden island")) OR AB ((australia* OR "new south wales" OR sydney OR queensland* OR Brisbane OR hobart OR tasmania OR "northern territory" OR darwin OR canberra OR aborig* OR "torres strait" OR victoria OR melbourne OR adelaide OR perth OR woodside OR Bungendore OR "Orchard Hills" OR Denman OR Berrimah OR "Pine Gap" OR "Port Philip" OR duntroon OR Holsworthy OR Adamstown OR "Mount Bundey" OR "Berry Springs" OR "Timber Creek" OR Cabarlah OR Canungra OR Enoggera OR Townsville OR Oakey OR "darling downs" OR "Shoalwater Bay" OR cairns OR "Cowley Beach" OR Glenorchy OR Launceston OR Devonport OR Warrane OR Richmond OR Burnie OR Albury OR Wodonga OR Puckapunya OR Swanbourne OR Karrakatta OR Lancelin OR Bindoon OR Karratha OR wagga OR Newcastle OR Katherine OR Weipa OR exmouth OR "garden island")) OR SU ((australia* OR "new south wales" OR sydney OR queensland* OR Brisbane OR hobart OR tasmania OR "northern territory" OR darwin OR canberra OR aborig* OR "torres strait" OR victoria OR melbourne OR adelaide OR perth OR woodside OR Bungendore OR "Orchard Hills" OR Denman OR Berrimah OR "Pine Gap" OR "Port Philip" OR duntroon OR Holsworthy OR Adamstown OR "Mount Bundey" OR "Berry Springs" OR "Timber Creek" OR Cabarlah OR Canungra OR Enoggera OR Townsville OR Oakey OR "darling downs" OR "Shoalwater Bay" OR cairns OR "Cowley Beach" OR Glenorchy OR Launceston OR Devonport OR Warrane OR Richmond OR Burnie OR Albury OR Wodonga OR Puckapunya OR Swanbourne OR Karrakatta OR Lancelin OR Bindoon OR Karratha OR wagga OR Newcastle OR Katherine OR Weipa OR exmouth OR "garden island"))
**S9**	(MH "Australia+")
**S8**	S4 OR S7
**S7**	S5 AND S6
**S6**	(MH "Community Reintegration") OR (MH "Retirement")
**S5**	(MH "Military Personnel+") OR (MH "Military Deployment+") OR (MH "War+")
**S4**	S1 OR S2 OR S3
**S3**	TI (((retired OR retire OR "ex-member*" OR reintegrat* OR transition*) N5 (militar* OR navy OR army OR navy OR sailor* OR solider* OR enlisted OR combat OR adf OR officer* OR "air force" OR "air-force" OR defense OR reservist* OR reserves OR trouper* OR "medical corp*" OR war OR wars))) OR AB (((retired OR retire OR "ex-member*" OR reintegrat* OR transition*) N5 (militar* OR navy OR army OR navy OR sailor* OR solider* OR enlisted OR combat OR adf OR officer* OR "air force" OR "air-force" OR defense OR reservist* OR reserves OR trouper* OR "medical corp*" OR war OR wars)))
**S2**	TI ((Veteran OR veterans OR "ex-serving" OR "ex-defense*" OR "Ex-service*" OR "ex-militar*" OR "past master*" OR "past-master*")) OR AB ((Veteran OR veterans OR "ex-serving" OR "ex-defense*" OR "Ex-service*" OR "ex-militar*" OR "past master*" OR "past-master*"))
**S1**	(MH "Veterans+")

Scopus

TITLE-ABS-KEY ((((veteran OR veterans OR "ex-serving" OR "ex-defense*" OR "Ex-service*" OR "ex-militar*" OR "past master*" OR "past-master*") OR ((retired OR retire OR "ex-member*" OR reintegrat* OR transition*) W/5 (militar* OR navy OR army OR navy OR sailor* OR solider* OR enlisted OR combat OR adf OR officer* OR "air force" OR "air-force" OR defense OR reservist* OR reserves OR trouper* OR "medical corp*" OR war OR wars))) AND (australia* OR "new south wales" OR sydney OR queensland* OR brisbane OR hobart OR

tasmania OR "northern territory" OR darwin OR canberra OR aborig* OR "torres strait" OR victoria OR melbourne OR adelaide OR perth OR woodside OR bungendore OR "Orchard Hills" OR denman OR berrimah OR "Pine Gap" OR "Port Philip" OR duntroon OR holsworthy OR adamstown OR "Mount Bundey" OR "Berry Springs" OR "Timber Creek" OR cabarlah OR canungra OR enoggera OR townsville OR oakey OR "darling downs" OR "Shoalwater Bay" OR cairns OR "Cowley Beach" OR glenorchy OR launceston OR devonport OR warrane OR richmond OR burnie OR albury OR wodonga OR puckapunya OR swanbourne OR karrakatta OR lancelin OR bindoon OR karratha OR wagga OR newcastle OR katherine OR weipa OR exmouth OR "garden island"))) AND (LIMIT-TO (PUBYEAR , 2022) OR LIMIT-TO (PUBYEAR , 2021) OR LIMIT-TO (PUBYEAR , 2020) OR LIMIT-TO (PUBYEAR , 2019) OR LIMIT-TO (PUBYEAR , 2018) OR LIMIT-TO (PUBYEAR , 2017) OR LIMIT-TO (PUBYEAR , 2016) OR LIMIT-TO (PUBYEAR , 2015) OR LIMIT-TO (PUBYEAR , 2014) OR LIMIT-TO (PUBYEAR , 2013) OR LIMIT-TO (PUBYEAR , 2012) OR LIMIT-TO (PUBYEAR , 2011) OR LIMIT-TO (PUBYEAR , 2010)) AND (LIMIT-TO (DOCTYPE , "ar") OR LIMIT-TO (DOCTYPE , "re"))

Cochrane Central
(((Veteran OR veterans OR "ex-serving" OR "ex-defense*" OR "Ex-service*" OR "ex-militar*" OR "past master*" OR "past-master*") OR ((retired OR retire OR "ex-member*" OR reintegrat* OR transition*) NEAR/5 (militar* OR navy OR army OR navy OR sailor* OR solider* OR enlisted OR combat OR adf OR officer* OR "air force" OR "air-force" OR defense OR reservist* OR reserves OR trouper* OR "medical corp*" OR war OR wars))) AND (australia* OR "new south wales" OR sydney OR queensland* OR Brisbane OR hobart OR tasmania OR "northern territory" OR darwin OR canberra OR aborig* OR "torres strait" OR victoria OR melbourne OR adelaide OR perth OR woodside OR Bungendore OR "Orchard Hills" OR Denman OR Berrimah OR "Pine Gap" OR "Port Philip" OR duntroon OR Holsworthy OR Adamstown OR "Mount Bundey" OR "Berry Springs" OR "Timber Creek" OR Cabarlah OR Canungra OR Enoggera OR Townsville OR Oakey OR "darling downs" OR "Shoalwater Bay" OR cairns OR "Cowley Beach" OR Glenorchy OR Launceston OR Devonport OR Warrane OR Richmond OR Burnie OR Albury OR Wodonga OR Puckapunya OR Swanbourne OR Karrakatta OR Lancelin OR Bindoon OR Karratha OR wagga OR Newcastle OR Katherine OR Weipa OR exmouth OR "garden island"))

Supplementary File - Table S2: Glossary of Outcome Measures

ADAS	Abbreviated Dyadic Adjustment Scale
AUDIT	Alcohol Use Disorders Identification Test
AQol	Assessment of Quality of Life
BAI	Beck Anxiety Inventory
BDI	Beck Depression Inventory
BDI-II	Beck Depression Inventory, second Edition
CAPS	Clinician Administered PTSD Scale
CAPS-5	Clinician Administered PTSD Scale-5 interview
CES-D	Center for Epidemiological Studies Depression Scale
CES	Combat Exposure Scale
CIDI	Composite International Diagnostic Interview
DASS21	Depression Anxiety Stress Scales
DAR	Dimensions of Anger Reactions Scale
DAR-5	Dimensions of Anger Reactions Scale - 5
EQ-5D	EuroQol Group
FAD	General Functioning subscale of the Family Assessment Device
GHQ-28	General Health Questionnaire
GSE	General Perceived Self-Efficacy Scale
GAF	Global Assessment of Functioning
GSI SCL	Global Severity Index of the Symptom Checklist
HAM-D	Hamilton Depression Rating Scale
HoNOS	Health of the Nation Outcome Scales
HADS	Hospital Anxiety and Depression Scale
IES	Impact of Events Scale
IES-R	Impact of Event Scale-Revised
IIP-32	Inventory of Interpersonal Problems 32-item version
PIH	Partners in Health
LSQ	Life Satisfaction Questionnaire
MARS	Mobile Apps Rating Scale
OHQ	Oxford Happiness Questionnaire
PCL-5	PTSD Checklist-5
PCL-M	PTSD checklist Military version
PNI	Positive and Negative Interactions Scale
P&G	Problems and Goals
Q-LES-Q-SF	Quality of Life, Enjoyment and Satisfaction Questionnaire-Short Form
SF-12	Medical Outcomes Study 12-Item Short Form Health Survey
SF-36	Medical Outcomes Study 36-Item Short Form Health Survey
SO-EAS	Social Functioning Assessment Scale
STAXI-2	State-Trait Anger Expression Inventory-2
STAI	State Trait Anxiety Inventory
SUDS	Subjective Units of Distress Scale.
WHODAS	World Health Organization Disability Assessment Schedule 2.0
uMARS	Mobile Apps Rating Scale user version
WHOQoL	World Health Organisation Quality of Life Instrument
WHOQoL-BREF	Brief World Health Organisation Quality of Life Instrument
WASAS	Work and Social Adjustment Scale
